# Supplementary material for: Segmentation-Free Estimation of Left Ventricular Ejection Fraction Using 3D CNN Is Reliable and Improves as Multiple Cardiac MRI Cine Orientations Are Combined
Source: Biomedicines. 2024 Oct 12;12(10):2324. doi: 10.3390/biomedicines12102324 (PMC11505352; doi:10.3390/biomedicines12102324)
Supplement: Supplementary file 1 [file biomedicines-12-02324-s001.zip › biomedicines-3220298-supplementary.pdf]

Supplementary Material:

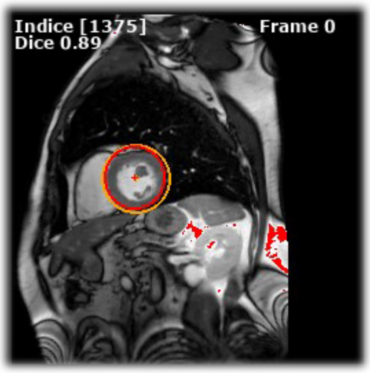

Supplementary Figure S1: Prediction of ellipsoidal ROI for the left ventricle: A classical VGG model was trained using the first 300 observations. The model predicts a tuple of four variables describing the ellipse: Cx and Cy (center coordinates), and Rx and Ry (radii). Red ellipse represent manual annotations, while orange ellipse indicate model predictions. The Dice coefficient was used to evaluate similarity between the manual (A) and predicted (B) ellipses, calculated as: Dice = 2×∣A∩B∣ / (∣A∣+∣B∣), where ∣A ∩ B∣ is the number of elements in the intersection of A and B, and ∣A∣ and ∣B∣ are the number of elements in sets A and B, respectively. The mean Dice coefficient obtained, after training, in the validation group was 0.89±0.05, suggesting that this model could be used in practice to expedite the pre-treatment step, although manual corrections may occasionally remain necessary.

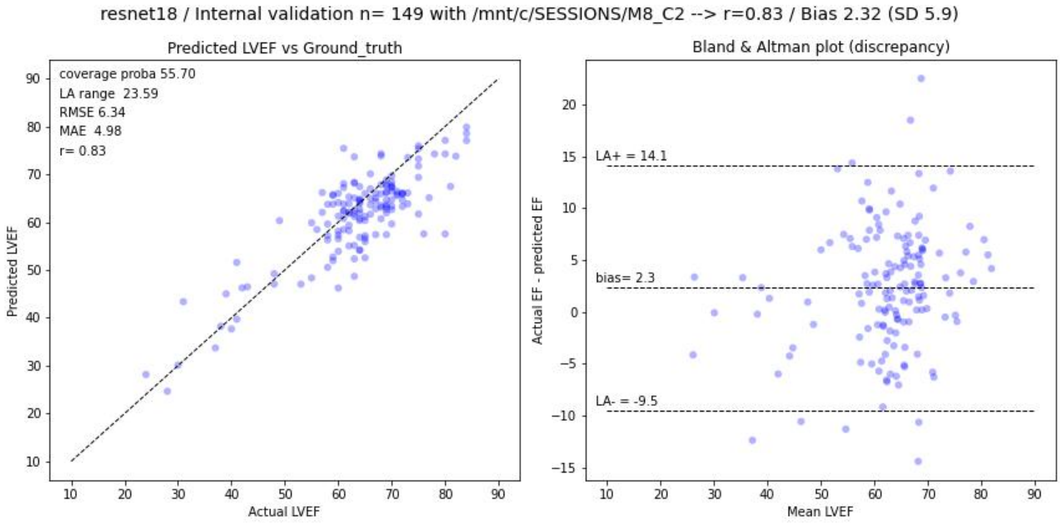

Supplementary Figure S2: Prediction of Left Ventricular Ejection Fraction (LVEF) based on two-long-axis (2LA) and six short-axis (SA) views in patients from the internal validation group demonstrated performance nearly identical to that observed in the 5-fold cross-validation experiment.

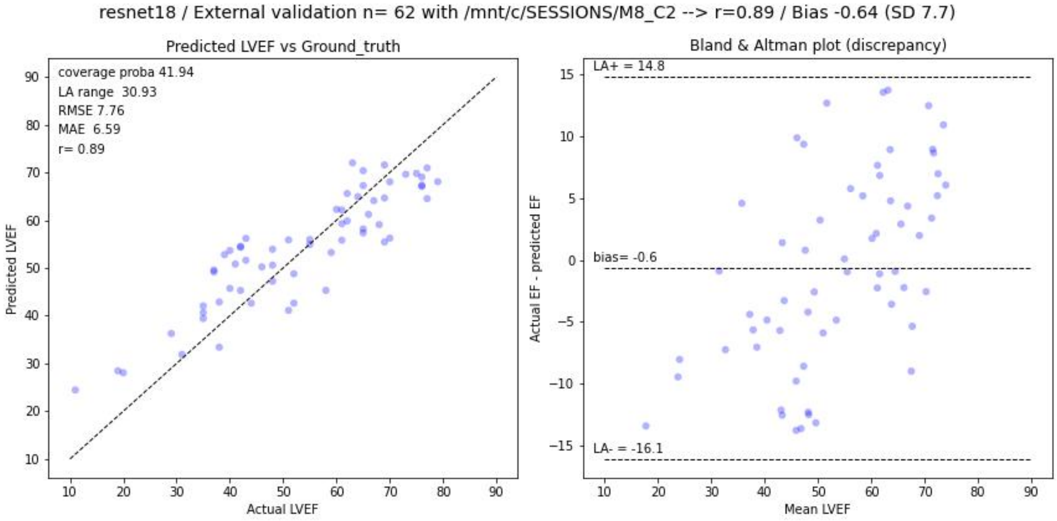

Supplementary Figure S3: Prediction of Left Ventricular Ejection Fraction (LVEF) based on two-long-axis (2LA) and six short-axis (SA) views in patients from the external validation group demonstrated lower performance as compared with the internal validation group.
